# Evaluation of the physician quality improvement initiative: the expected and unexpected opportunities

**DOI:** 10.1186/s12909-015-0511-2

**Published:** 2015-12-22

**Authors:** Kirsten Wentlandt, Andrea Bracaglia, James Drummond, Lindsay Handren, Joshua McCann, Catherine Clarke, Niki Degendorfer, Charles K. Chan

**Affiliations:** Department of Family and Community Medicine, Division of Palliative Care, University of Toronto, Toronto, ON Canada; Medical Affairs, University Health Network, Toronto, ON Canada; School of Public Policy and Governance, University of Toronto, Toronto, ON Canada; Human Resources, University Health Network, Toronto, ON Canada; Corporate Planning, University Health Network, Toronto, ON Canada; Division of Respirology, Department of Medicine, University of Toronto, Toronto, ON Canada; Toronto General Hospital, UHN, 9NU-925, 200 Elizabeth St, Toronto, ON M5G 2C4 Canada

**Keywords:** Multi-source feedback, Physicians, Clinical competence, Accountability, Quality improvement

## Abstract

**Background:**

The Physician Quality Improvement Initiative (PQII) uses a well-established multi-source feedback program, and incorporates an additional facilitated feedback review with their department chief. The purpose of this mixed methods study was to examine the value of the PQII by eliciting feedback from various stakeholders.

**Methods:**

All participants and department chiefs (*n* = 45) were invited to provide feedback on the project implementation and outcomes via survey and/or an interview. The survey consisted of 12 questions focused on the value of the PQII, it’s influence on practice and the promotion of quality improvement and accountability.

**Results:**

A total of 5 chiefs and 12 physician participants completed semi structured interviews. Participants found the PQII process, report and review session helpful, self-affirming or an opportunity for self-reflection, and an opportunity to engage their leaders about their practice. Chiefs indicated the sessions strengthened their understanding, ability to communicate and engage physicians about their practice, best practices, quality improvement and accountability.

Thirty participants (66.7 %) completed the survey; of the responders 75.9, 89.7, 86.7 % found patient, co-worker, and physician colleague feedback valuable, respectively. A total of 67.9 % valued their facilitated review with their chief and 55.2 % indicated they were contemplating change due to their feedback. Participants believed the PQII promoted quality improvement (27/30, 90.0 %), and accountability (28/30, 93.3 %).

**Conclusions:**

The PQII provides an opportunity for physician development, affirmation and reflection, but also a structure to further departmental quality improvement, best practices, and finally, an opportunity to enhance communication, accountability and relationships between the organization, department chiefs and their staff.

## Background

Multi-source feedback (MSF), or 360-degree evaluation, is an expanding approach to the assessment of physicians. A survey based process using self-assessment and physician colleague, co-worker and/or patient reviewers, MSF can be a valuable method to assess residents and physicians [1–5] and has previously encouraged practice improvement [6, 7]. MSF has successfully been used as both a formative assessment and as a quality improvement approach to drive advancements in performance and medical education [8, 9].

The Physician Quality Improvement Initiative (PQII) was a project initiated by the Council of Academic Hospitals of Ontario to provide active physicians, in collaboration with their physician department chiefs, comprehensive feedback that can be used as a guide for quality improvement in their practice. Utilizing the well-established Physician Achievement Review (PAR) program [2, 7, 10], the PQII incorporates an additional step; a facilitated feedback review with their department chief. This initiative was seen as an opportunity to encourage improvements to physician practice and to engage physicians in a quality improvement agenda. As one of the PQII pilot sites, University Health Network (UHN) implemented the PQII in several medical and surgical departments and then underwent a comprehensive project evaluation.

The purpose of this current mixed methods study is to examine the value of the PQII by eliciting feedback from key stakeholders within the organization. The study addressed the following questions: (1) What themes and subthemes emerged from a qualitative analysis of the department chief’s and participant physician’s feedback on the PQII implementation and project outcomes? (2) What was the frequency of the intended outcomes identified by the participant physicians?

## Methods

University Health Network conducted an evaluative study of Physician Quality Improvement Initiative, an MSF process using the standardised Physician Achievement Review (PAR) tools, and facilitated report review.

### Study background

The MSF process utilizing the PAR surveys has been published elsewhere and will be briefly summarized here [2, 7, 10]. Volunteer physician participants identified at least eight medical colleague and eight co-worker reviewers, and 18 patient reviewers. General domains of interest were surveyed using a 5-point Likert scale, and they included: communication, collegiality, professionalism, clinical performance and office management. Physician participants (PPs) completed a self-assessment questionnaire. Feedback was compiled in the form of a report, which provided individual and aggregate mean scores by domain and individual scores for items within each domain.

The report is reviewed with participants in a feedback review session organized by their department chief (DC). This session is utilized to review the feedback, help contextualize, and support the participant’s development and utilization of the material in a meaningful way. There is also an opportunity to initiate a development plan to support improvement or leverage strengths. This is discordant from the original PAR program, which mails participants their report and allows for self-directed analysis and action for development. DCs were supplied with coaching tools and met with the PQII project lead to review their group’s reports to help strategize optimization of meeting structure and feedback for the report reviews.

### Study design

All PPs who completed the pilot of the PQII (*n* = 45) were invited to provide feedback on the project via online survey and/or an interview. DCs were invited to participate in an interview. Interviews utilized open-ended and guided questions to elicit participants’ descriptions of experiences and perceptions around their participation in the PQII. Interviews lasted approximately 1 h and were audio recorded and transcribed verbatim.

The online survey consisted of 12 questions that focused on physician’s perception of the project’s promotion of quality improvement and accountability, the value of the project and it’s components and if they were considering any changes to their practice based on their feedback. These were scored as agreement or disagreement with the statements. The pilot project began in January 2013 and evaluation of the project was completed by June 2014. University Health Network’s Research Ethics Board (REB) was consulted and official REB approval was deemed not necessary. This work was carried out in accordance with the Declaration of Helsinki, including but not limited to the guaranteed anonymity and informed consent of all participants.

### Data analysis

We conducted the analysis as a team, using accepted analytical procedures for qualitative data [11]. First, using a content analysis approach, two transcripts were reviewed and coded, and discussion led to the development of a coding framework. This framework was used to analyse the remaining transcripts and discussion of emerging themes and revision of the coding structure was done as required. Data was compared and contrasted within and among participants and themes, to determine and interpret relationships and confirm dominant themes [12].

## Results

A total of 5 DCs and 12 PPs participated in the semi structured interviews. Two of the DCs and four of the PPs were from departments in surgery. The remaining participants were from varied medical departments. Three of the participants interviewed were women.

### Perceptions of appropriateness, and purpose of the MSF process

PPs indicated they understood and recognized the relevance of the project for the physician population. It was ‘accepted there’s a need to evaluate physicians’ (PP7) and overall ’a good initiative for doctors’ (PP6). PPs agreed that the global purpose of this review process was for practice enhancement and quality improvement. The prospect for performance feedback was seen as an opportunity for their own personal improvement but PPs were skeptical of the improvement of others (Table 1). DCs also recognized the limitations to affect change through the feedback process and were interested in building the PQII into a more global strategy for quality improvement and culture change.

**Table 1:** Perception of the MSF process

Multisource feedback
*Perceptions of appropriateness, and purpose of the MSF process*: PPs indicated they understood and recognized the relevance of the project for the physician population. The prospect for performance feedback was seen as an opportunity for their own personal improvement but PPs were skeptical of the improvement of others.
*I’m a big supporter of these projects. I think if we don’t assess ourselves we will not know about our performance. That’s one way of assessing ourselves and maybe in the future we’ll find out that we need to tweak it a little bit. PP2*
*I think that these questionnaires, these sorts of interrogation tools are very useful for gathering information. It really is dependent upon other people as to how much you effect changes. If you received information through a questionnaire such as this that suggests that somebody is under-performing, that gets you information about that performance, but not necessarily ensures that something will be then done … We hear the fire alarm go off every day. It doesn’t necessarily mean that we are going to have less fires; it just means that you can hear the fire alarm. PP1*
*Culture change*: The need to build the PQII into a more global strategy for quality improvement and culture change.
*I think the whole shift has to be really about creating a culture where people actually care what the answers are. A lot of what we’ve talked about today is about the mechanics of asking people a question and the mechanics of giving them the answer. What you have to do first and foremost is make people care about what the answer is. If people care about what the answer is they’ll find out the information in a variety of ways. DC2*
*Barriers to completing the MSF process:* Physicians had varied success collecting reviewer feedback. Other limitations discussed by participants included finding the time to sign in to the online program and select reviewers, the response rate of physician colleague and coworker reviewers and manually adding contact information of reviewers who worked outside of the organization.
*I don’t work with eight doctors. I know eight doctors. I had to scramble. I only work with about three or four people in an intimate way, that constant interaction, constant work. PP7*
*Receiving Feedback* **:** Participants were engaged and invested in receiving their feedback. PPs and DCs indicated that the feedback affirmed that overall the participants were doing a good job, with few exceptions. PPs found the process reassuring, useful and an opportunity for self-reflection and to contemplate possible improvements.
*I have a pretty good idea of how I practice already and I know I’m… what it did is it actually reinforced that I’m a pretty good read of my own strengths and my own weaknesses based on… in that sense, it was a useful exercise…… I think it can be very enlightening. It’s reassuring to know that at the end, what I think of my practice is what other people think of my practice. That was comforting. PP9*
*I remember… it’s nice to have the averages and see where you sit within the cohort, like if you’re the middle or above. I found that actually quite useful. PP9*
*It reinforces that you actually are doing a reasonable job. That’s an important thing, because it can reaffirm that. PP6*
*“That’s an area to improve. How do I improve it?” Now I would have to go to my patients and say, “How can I improve?” Maybe that’s what I should do. Maybe that’s up to me. PP8*

*PQII* Physician Quality Improvement Initiative, *PP* Participating Physician, *DC* Department Chief

### Difficulties in completing the MSF process

Reviewers’ ability to appropriately assess physicians effectively was central to participants’ concerns about recruiting medical colleague and co-worker reviewers. Practice design played a significant role. In departments where physicians shared patients, had a multidisciplinary team and had an outpatient clinic, selection of reviewers was seen as ‘straightforward’ (PP9), ‘pretty reasonable’ (PP6) and ‘not hard’ (PP10). Participants that worked in isolation, strictly on inpatient units, and had smaller teams found collection of data quite ‘challenging’ (PP2), ‘frustrating’ (PP4) and ‘excessive’ (PP2).

Other limitations discussed by participants included finding the time to sign in to the online program and select reviewers, the response rate of physician colleague and co-worker reviewers and manually adding contact information of reviewers who worked outside of the organization.

### Receiving feedback

Participants were engaged and invested in receiving their feedback. PPs and DCs indicated that the feedback affirmed that, overall, the participants were doing a good job, with few exceptions. PPs found the process reassuring, useful and an opportunity for self-reflection, affirmation and to contemplate possible improvements.

### Guided review session

#### Preparation for meeting

Some DCs were unsure of how to manage the facilitation of the report review sessions and expected difficulties with select groups of physicians. The chiefs were reassured and gained confidence using the coaching documents and tools, as well as meeting with the project lead to discuss facilitation strategies. A summary of quotes can be found in Table 2.

**Table 2:** Preparation for the review session

Preparation for facilitated review session
Some DCs were unsure of how to manage the facilitation of the report review sessions and expected difficulties with select groups of physicians. The chiefs were reassured and gained confidence using the coaching documents and tools, as well as meeting with the project lead to discuss facilitation strategies.
*How do you get physicians to open up in a very candid and honest way about the feedback that is not so positive, rather than just say “oh yes, I’m going to improve.” It’s a difficult conversation. That’s really the most difficult part of the entire process, right, is where you try and spin it as positively as you can, and it should be a positive experience. Ultimately, the people that are the most problematic, are usually the people that are the least likely to tolerate the criticism. DC3*
*It was useful as a way of framing it, the way of dealing with more perhaps critical feedback, how to frame that and pitch it, really, and work through it, developing a prescription, really, to improve. DC5*
*Once you walked through the process, which was great, the process had clear outlined expectations of how to use the document. It came with some resource to walk the individual through how to make some change and some choices. Having been through some coaching and an interest in mentoring and coaching and sponsorship myself, it resonated exactly with how I would approach this. It fit for the stuff that I’ve done in the past. When I walked through it, it was very helpful. It made the process quite easy. DC1*
*I think that the debriefing afterwards in preparation for providing the feedback is probably one of the most important things. DC4*

*DC* Department Chief

#### The meeting session

PPs indicated they found the ‘dissection of the results was helpful’ (PP10), and ‘valuable’ (PP6). The report review session was found to be ‘relevant’ (PP5), and viewed as an opportunity to have a face-to-face meeting and discuss personalised objectives with their DC. DCs also felt that the participants responded well to the review session and that, overall, the experience was a positive one (Table 3).

**Table 3:** The facilitated review session

Facilitated Review Session
*Overall Impression:* PPs indicated they found the facilitated meeting valuable and the ability to go through their results with their DCs as helpful. It was seen as an opportunity to discuss their interests and possible career goals. DCs also felt that the participants responded well to the review session and that overall the experience was a positive one.
*After a while we stopped talking about it, had probably half an hour to talk about what are my medium and longer-term objectives, what’s working? What’s not working? What are am I happy about, and so on? It was, I think, a very good experience in that sense. PP2*
*I asked them if they felt it was worthwhile and they all did. DC5*
*It was a nice opportunity. Some people were very receptive for feedback. They welcomed it. They viewed that as a way to move forward. There’s a couple of people in my division who really believe that. I think people were interested on their marks or how they scored and how they’re perceived. DC5*
*Setting Goals:* DCs struggled to facilitate development for physicians with strongly positive feedback and DCs felt more equipped to work with physicians that had clear areas for improvement but questioned the authenticity of the conversation and true engagement those physicians who received negative feedback.
*With the outstanding one, we found it very hard to develop a plan to perpetuate his outstandingness; whereas, I think for the one that had more criticism could reflect on the criticism- this is what I have to do to improve. What I have to do to sustain versus what I have to do to improve. DC3*
*For some of the good ones…It was just time-wise. I think some people didn’t see the value in it. DC5*
*I find that sometimes, as a defence, physicians can sometimes be overly conciliatory and polite and agreeable as a way of maybe a defence of being reflectively honest, an honest reflection. I don’t know if I was prepared for that. I’m not sure if I have the skills or not, in how to get a physician to be more open. DC3*
*Building Relationships and Understanding*: DCs found the process strengthened their understanding, was found to be relationship building. DCs indicated the report review was an opportunity to begin a conversation about areas for improvement that they were unable to engage in previously.
*In that sense, I found the coaching sessions to be quite good. It’s not my comfort zone to be like I’m the boss and you should do what I do or say or anything. My divisions are all very, very high performing people. It’s really not my place to tell them how to do a good job, especially when they’re all above average or superstars. It’s a little bit different dynamic of trying to guide or explore things with people. In general, I found it a very positive experience. DC2*
*I think it has positively affected. I think they got a sense that I was genuinely interested in how they’re doing. I certainly told them that I want to be able to help them as professionals and that that’s the purpose. Certainly I don’t often meet with them one on one, so three meetings one on one, it enhanced our relationship. I think it was positive. DC3*
*I think it affected my relationship with the staff in a good way. I think that ideally we should meet with the staff on a regular basis…It’s rare to have a conversation which is about what are your longer-term objectives; what’s going on in your life and so on? … We actually had time. DC2*
*The one negative exploration I had came about with respect to the single faculty member who scored on the self-assessment of lower score in something. I used that to begin a conversation about an area that I thought that that faculty member was not strong in myself. Regardless of what they thought, I thought they weren’t that good in that area. I said, ‘Oh, you marked yourself low. Tell me about that. What do you think? What can I do to help?’ It was a way to bring up a subject, which actually I was somewhat tentative to bring up in any other circumstance or with any other introductory context. DC1*
*Accountability*: DCs indicated that the PQII allowed for introduction of oversight that did not exist previously within the organization.
*I’ve given them feedback over the years, because we have a re-appointment process. It’s usually been absence of negative feedback is all that’s been there. There were no complaints against you this year, as opposed to, and there were maybe one or two accolades that came along to the program that may have identified you or may not have identified you. We really didn’t have a robust method. …. It’s nice to see that we’ve got an accountability process. DC1*
*I think it is important that there is some oversight and that people really realize that this kind of, the perceptions of your colleagues and of your patients is going to be taken into account and that there are ways to improve physician behaviour. Sometimes it has to be done. DC4*
*I think it also puts individuals on notice that it’s not just me looking over their issues; there’s some sort of third party hospital, there’s some officialness. Even though we don’t work for the hospitals, we have to apply for privileges every year and this is part of the credentialing process. Knowing that they’re looking at it allows you, because otherwise you’re self-employed, you’re self-disciplined. Self-discipline is good, but it’s not good for everybody. Some people need more. They need Big Brother occasionally. DC2*
*Department-Wide Initiatives*: The PQII gave DCs the ability to review the department as a whole, evaluate strengths and weakness and consider implementation of department wide initiatives to improve care. Identification and leverage of physician’s strengths were seen as an opportunity for knowledge translation and transferring best practices.
*The things that we talked about were, number one, about the positive things that people got good scores on. We would try and figure out if there’s something in that good score that we could do more of as a division that other faculty people could do. For example, one of my faculty got a good score for communication with patients. We talked about how he draws a diagram for every single patient, no matter how complicated or simple the operation is and gives it to them. I relayed that story to one of my other faculty members. He said, ‘Oh I always do a diagram too, but I keep it in the chart.’ We discussed the relative benefits of that and so on. I think it was helpful as a way of transferring best practices between people. DC2*

*PQII* Physician Quality Improvement Initiative, *PP* Participating Physician, *DC* Department Chief

DCs struggled with making the review session meaningful, and facilitate development for physicians with strongly positive feedback. DCs felt more equipped to work with physicians who had clear areas for improvement but indicated that not all participant interviews went well for those with negative feedback. One DC wondered about the authenticity of the conversation and true engagement of a physician who received negative feedback.

DCs found the process strengthened their understanding, was found to be helpful in relationship building, and a learning experience for both them and their staff. It was seen as an opportunity that did not exist before to support their staff; towards improvement but also to further their staff’s personal goals. DCs indicated the report review was an opportunity to begin a conversation about areas for improvement they were aware of prior to the PQII but were unable to engage in previously.

Having the space to freely discuss feedback and facilitate the organization of a development plan was seen as an advantage to further accountability within their department. Department chiefs indicated current processes were ineffectual to promoting responsibility and accountability of physicians and that the PQII allowed for introduction of oversight that did not exist previously within the organization.

Finally department chiefs recognized that the PQII gave them the ability to review the department as a whole, evaluate strengths and weaknesses and consider implementation of department-wide initiatives to improve care. Indications to improve shared practices and resources such as clinic space and patient resources were found. In addition, identification and leverage of physicians’ strengths were seen as an opportunity for knowledge translation and transferring best practices.

### Quantitative survey

A total of 30 PPs completed the exit survey for a completion rate of 66.7 % (30/45). A summary of results is depicted in Table 4. Of the responders 89.7 % (26/29) found co-worker feedback valuable and 67.9 % (26/29) valued their facilitated review session with their department chief. A total of 55.2 % (16/29) of physicians indicated they were contemplating a practice change in regards to the feedback, and the majority of these physicians (12, 75.0 %) planned to change how they communicate with patients. Ninety percent of responders believed the PQII promoted quality improvement, and 93.3 % accountability.

**Table 4:** Online survey results

Item	Agree
The PQII promotes quality improvement	27/30 (90.0 %)
The PQII promotes accountability	28/30 (93.3 %)
Participation in the PQII project was easy	10/30 (33.3 %)
Feedback from patients was valuable	22/29 (75.9 %)
Feedback from co-workers was valuable	26/29 (89.7 %)
Feedback from other physicians was valuable	26/30 (86.7 %)
My meeting with my department head was valuable	19/28 (67.9 %)
I have contemplated practice changes as a result of this project	16/29 (55.2 %)
Based on my PQII feedback I have made changes in…	
My communication with patients.	12/28 (42.9 %)
Aspects of patient care (e.g. investigations, education, prevention)	9/28 (32.1 %)
How I work with co-workers	7/28 (25.0 %)
How I interact with medical colleagues	6/28 (21.4 %)

Note: Not all the participants answered all questions

*PQII* Physician Quality Improvement Initiative

## Discussion

In this study we utilized an established MSF tool and a facilitated feedback review session between physicians and their chiefs to target physician development and performance improvements. This is the first time in the literature that these tools have been used in an organizational (vs regulatory body) quality improvement initiative, with the use of a report review session done in partnership with physician leaders. This project evaluation provides promising data that indicate PQII provides an opportunity for individual physician development, affirmation and reflection, but also a structure to further departmental quality improvement, transfer of best practices, and finally, an opportunity to enhance communication, accountability and relationships between the organization, department chiefs and their staff.

Participants indicated that the MSF practice was a valuable process that promoted quality improvement. Overall 54 % contemplated practice changes in response to their PQII feedback. These changes were most often related to how they communicate with patients. This is in line with findings from the PAR program where in 2011, 70 % of Alberta physicians felt that PAR feedback was valuable, and 40 to 50 % reported that they had made changes in at least one aspect of practice, most often in aspects of direct patient care and communication [13].

Feedback is often described as the act of providing knowledge of the results of behaviour or performance to the individual [8, 9]. However, feedback is more than simply providing information, and must include an action to close the identified gap and promote improvement [9]. Although the PAR MSF process helps to inform practice improvements [6, 7, 14], it is clear that the success of MSF is dependent on many factors. These include participant’s emotional reactions to the feedback, the congruence between the feedback and their personal beliefs about themselves, and the nature and characteristics of the feedback itself [15, 16].

Evidence demonstrates that the extent to which practice changes occur following provision of MSF will be influenced by the acceptability of perceived negative information [17] and the approach in which motivation is encouraged [18]. The goal of the PQII facilitated review session is to have the DC help participants translate their feedback in a meaningful way to encourage development. This meeting also facilitates DC understanding of their frontline staff and it naturally extends into the relevance of the feedback towards career goals and how the DC and their department can support the physician’s development. The majority of PPs in this project indicated they found this session valuable and an avenue to garner support for development and career goals.

Facilitating these report review sessions with their physicians improved familiarity, understanding and communication with their staff. The PQII mandated this report meeting, to engage their staff around their clinical practice and future plans- an opportunity often lacking due to time and competing activities. Also, recognizing that individual physician performance problems may identify larger systems challenges, the PQII also allowed DCs to aggregate information to understand particular patterns of strength and weakness in performance within the department and organization. In this study, department chiefs indicated they were able to identify best clinical practices and evidence-practice gaps. Knowledge of these opportunities allows for the development of physician-led quality improvement programs which can be shared more widely [19].

Finally, the PQII has been seen as an opportunity by DCs to engage physicians around their accountability in providing quality care. Accountability has been highlighted as an important element of a safe and quality care culture in high performing healthcare systems [20]. A great number of hospital-based physicians are independent professionals, reimbursed by the government and not considered employees of the organization or university. Often their only true link to the health care organization or their academic centre is through the reappointment or credentialing process that is overseen by a physician leader or department chief. Accountability can be difficult to connect to this annual privilege review as it is frequently a perfunctory process and is rarely linked to performance [21]. By investing in the PQII, the organization demonstrated its support and interest in physician quality improvement and the DCs in turn were able to engage physicians on the subject of their accountability to their department and organization [22]. Preparation is imperative to support the implementation of data collection but also to ensure DCs’ ability to facilitate a useful and meaningful report review.

This project has many limitations. There may be selection bias as these participants are physicians who volunteered to complete the PQII and are predisposed to think positively about the project. The project was a pilot study and has a small sample size. Several participants had faced significant barriers to completing the PQII, and this may have influenced the perceived usefulness and value of the project. Future PQII implementation should include strategies to minimize these barriers to ensure future engagement of project objectives and outcomes. Future studies could explore the influence of the department chief’s facilitated review session on the perception of physicians’ feedback, as well as determine the most effective method to provide feedback to enhance practice improvements and continued learning.

## Conclusion

Utilizing a well-established MSF program and introducing a facilitated report review session with their department chiefs, the PQII provided a novel opportunity to supply physicians with valuable performance feedback. This project evaluation provides promising data in support of the PQII as an opportunity for individual physician development, affirmation and reflection, in addition to the provision of other, unexpected opportunities including a structure to further departmental quality improvement, transfer of best practices, and finally, an opportunity to enhance communication, accountability and relationships between the organization, department chiefs and their staff.
